# Community Health Dialogue Implementation for the Solution of Loneliness in Rural Communities: Autoethnography

**DOI:** 10.7759/cureus.67245

**Published:** 2024-08-19

**Authors:** Ryuichi Ohta, Toshihiro Yakabe, Hiroshi Adachi, Chiaki Sano

**Affiliations:** 1 Communiy Care, Unnan City Hospital, Unnan, JPN; 2 Family Medicine, Unnan City Hospital, Unnan, JPN; 3 Community Medicine Management, Faculty of Medicine, Shimane University, Izumo, JPN

**Keywords:** primary care, general medicine, family medicine, qualitative research, aged, rural health, community health services, loneliness, social isolation

## Abstract

Introduction: Isolation and loneliness among older adults in rural communities pose significant risks to physical and mental health, leading to higher rates of morbidity and mortality. This study investigates the impact of continual rural health dialogues facilitated by family physicians on reducing loneliness and enhancing community health in Unnan City, Shimane Prefecture, Japan.

Method: Using a constructivist grounded theory approach, we conducted a qualitative study involving 165 participants over 65 from five rural communities between April 2022 and March 2024. Monthly health dialogues covered chronic diseases, exercise, and polypharmacy. Data were collected through ethnographic observations, focus group interviews, and field notes, with iterative coding and analysis to identify themes and concepts.

Results: Three primary themes emerged: the existence of loneliness and its impact on health, motivation to address loneliness through a sense of security, and recognition of the importance of community engagement in reducing loneliness. Participants reported increased health awareness, enhanced community interaction, and recognition of loneliness's prevalence and health impacts. Regular dialogues fostered trust with healthcare professionals, encouraged proactive health management, and facilitated supportive community connections. These interactions significantly reduced feelings of loneliness and improved health outcomes.

Conclusion: Continual rural health dialogues effectively mitigate loneliness and enhance health outcomes in rural communities by fostering regular interactions and building supportive networks. These findings underscore the importance of community engagement and continuous relationships with healthcare professionals in addressing loneliness. Policymakers and healthcare providers should consider integrating such dialogues into rural health strategies to promote healthier, more connected communities. Future research should explore these interventions' long-term sustainability and broader applicability across diverse rural settings.

## Introduction

Isolation and loneliness are critical issues in aging societies, where a significant portion of the population experiences reduced social interaction and emotional support, with a prevalence of social prevalence of 24% among people over 65 years old [1,2]. These conditions are not just a matter of emotional well-being but have profound implications for physical health [3]. Social isolation and loneliness can lead to a range of physical and mental health issues, including depression and cardiovascular diseases, which in turn increase the risk of higher mortality rates among older adults [4].

Public and private organizations have launched community outreach programs, social support groups, and volunteer-driven activities to enhance community interaction, reduce isolation, and provide concrete support for those affected. Interestingly, previous reports indicate that it is not merely the frequency of personal interactions that alleviates loneliness but the level of community participation [3,5]. Regular participation in community activities, such as neighborhood clean-up projects, local gardening groups, and social clubs, has been linked to reduced loneliness, indicating that a sense of belonging and active involvement in communal life can significantly enhance the well-being of older adults [6]. Consequently, improving the conditions of loneliness can lead to better overall health outcomes for older people, thereby strengthening the entire community's health [7].

Rural health dialogues driven by family physicians are one promising approach to fostering community participation [8]. These dialogues involve community members gathering at local centers to discuss health-related topics and community issues with family physicians [9]. This method addresses individual health concerns and brings isolated individuals together, facilitating their integration into the community [8,9]. By participating in these dialogues, isolated individuals can express their concerns and feel a sense of belonging, which is crucial in mitigating loneliness and social isolation.

The continual provision of rural health dialogue offers a sustainable solution to the pervasive issues of isolation and loneliness in rural communities. By regularly organizing these dialogues, community members can maintain ongoing engagement with each other and healthcare professionals [10]. This continuous interaction helps build more robust social networks and provides a consistent support system for those who are isolated. Moreover, addressing community issues through these dialogues can lead to more cohesive and resilient communities.

This study aims to clarify the effect of the continual provision of rural health dialogues on community isolation and loneliness. By examining the impact of these regular interactions, the study seeks to understand how sustained community engagement through structured dialogues can alleviate the adverse effects of isolation and improve the overall health of older adults in rural settings. Through this research, we hope to provide evidence-based insights that inform the development of effective interventions to combat loneliness and promote healthier, more connected communities.

## Materials and methods

This qualitative study employed a grounded theory approach to explore changes in the perception of loneliness among participants involved in rural health dialogues in rural communities. By adopting a constructivist grounded theory framework, our decisions and analyses were guided by an emphasis on co-creating knowledge between the researcher and participants. This approach, which integrates the researcher’s reflections and interactions with participants as part of the data, allowed for a more profound understanding of participants' perceptions of loneliness in rural settings. Consequently, this theoretical perspective facilitated the development of a rich, contextually grounded understanding of how engaging in rural health dialogues impacts the perception of loneliness, recognizing the varied and complex experiences of the participants.

Setting

The study was conducted in Unnan City, Shimane Prefecture, Japan. Positioned in the eastern part of Shimane and bordering Hiroshima Prefecture to the south, Unnan City covers a land area of 553.1 km², representing 8.3% of Shimane Prefecture. The landscape is predominantly forested, emphasizing its rural character and a considerable distance from urban centers.

According to a 2020 survey, Unnan City had a population of 36,007, with 40.01% of residents over 65 years old. This significant elderly population is typical of rural areas and reflects the city's social organization. Unnan comprises 30 multifunctional autonomous communities, each addressing various social issues and needs indicative of demographic trends and social structures common in rural Japan [11].

Politically, Unnan City operates within Shimane Prefecture's local governance framework, which prioritizes community participation and citizen empowerment. Local authorities manage public services, including healthcare, through various community organizations, highlighting the city's unique political and social structure.

Regarding healthcare, Unnan City encounters challenges common to rural areas, such as limited access to healthcare facilities and professionals. Residents frequently depend on local clinics and healthcare centers and may need to travel to neighboring areas for specialized medical services. The limited availability of healthcare professionals means that visiting practitioners are often relied upon for specific medical needs [11].

Participants

Between April 2022 and March 2024, 165 citizens from rural communities participated in rural health dialogues. Purposive sampling was employed to meet the research objectives of conducting ethnographic and semi-structured interviews. These dialogues were held in five communities (Tane, Matsukasa, Daito, Mitoya, and Kuno), which had a combined population of 3,268 as of May 2023 [12].

The inclusion criteria for participants were residency in the communities, being 65 years or older, the ability to provide informed consent, and willingness to participate in the study. Exclusion criteria included non-residency in Tane and Matsukasa, under 65 years old, inability to provide informed consent, or unwillingness to participate. Participants were briefed about the study at their community centers and provided informed consent according to these criteria.

Rural health dialogue

A rural health dialogue was organized among family physicians and older residents to exchange experiences and knowledge regarding health-seeking behaviors (HSBs) in rural communities. These health dialogues occurred monthly at each community center, with 10-12 participants in each session [8]. Each session focused on a specific health theme, such as chronic diseases (hypertension, dyslipidemia, diabetes, and chronic obstructive pulmonary diseases), exercise, nutrition, sleep, joint pain, constipation, polypharmacy, multimorbidity, and palliative care. The sessions lasted between 60 and 90 minutes.

During each health dialogue, family physicians presented a health topic, discussing the prevalence of diseases in the community, related conditions, treatments, and specific experiences with these diseases. Following the presentation, participants voluntarily shared their own health experiences. Family physicians encouraged this sharing and addressed any questions that arose. Based on participants' experiences, physicians inquired about their actions regarding symptoms and HSBs. Various strategies and behaviors for managing symptoms were discussed, with physicians respecting each participant's approach and the reasons behind their behaviors. Finally, family physicians shared examples of effective behaviors to manage symptoms and prevent worsening conditions [9].

These dialogues often revealed new health issues. Family physicians facilitated discussions on these new topics, delving deeper into how health issues develop within the community and exploring potential approaches. In most cases, solutions were developed during the sessions, prompting participants to consider concrete actions and discuss them with friends and family until the following rural health dialogue.

Measurements

Ethnographic and Focus Group Interviews

Observational and focus group interviews, aligned with grounded theory methodology, were conducted with the participants [13]. The observations aimed to understand the context and interactions among community center participants, informing the subsequent focus group interviews. Although ethnographic methods inspired our observational strategies, they primarily enhanced our grounded theory approach [14]. These observations provided rich contextual data that were instrumental in developing our interview guide and deepening the focus group discussions. The ethnographic study included only rural citizens.

The primary researcher (RO) specialized in family medicine, medical education, rheumatology, and public health. Researchers in rural clinics observed participant interactions at community centers and took detailed field notes. RO facilitated the focus group interviews following each health dialogue session in the community center room. The interview guide comprised three questions: How does the rural health dialogue with family physicians impact your community’s health? What are the advantages of rural health dialogues? Do you have any suggestions for improving the dialogue on rural health? Each interview lasted about 60 minutes and was recorded and transcribed verbatim.

Analysis

Using an inductive grounded theory approach, this study explored how participants in rural health dialogues altered their perceptions and behaviors concerning HSBs and loneliness [15]. The first researcher began by thoroughly reading the field notes and conducting in-depth semi-structured interviews, coding the content to develop initial codebooks based on repetitive readings. The process and concept coding methods were employed [16]. This involved iteratively inducing, merging, deleting, and refining concepts and themes through constant comparison with the research materials during the initial coding phase, leading to axial coding. Axial coding further elaborated on and refined the concepts and themes.

For triangulation, the first and second researchers collaboratively discussed the emerging concepts and themes. The content of the interviews was analyzed iteratively throughout the research period to ensure theoretical saturation. Finally, both researchers reviewed and agreed upon the theory, leading to the identification of the final themes.

Ethical consideration

Participant anonymity and confidentiality were ensured throughout the study. All participants provided written informed consent before participating in the rural health dialogues. All procedures complied with the Declaration of Helsinki and its subsequent amendments. The Unnan City Hospital Clinical Ethics Committee approved the study protocol (No. 20230010).

## Results

The grounded theory approach developed three themes: the existence of loneliness and its impact on health, motivation to address loneliness through a sense of security, and recognition of the importance of community engagement in reducing loneliness. The rural health dialogues significantly enhanced participants' community interaction and health awareness, leading to several notable outcomes. Participants gained new health knowledge, which they shared within their communities, fostering increased communication and addressing previously unrecognized health concerns. The dialogues helped participants recognize the prevalence of loneliness, even in rural areas, and the associated health impacts, prompting a community-wide response to mitigate these issues. Regular interactions with family physicians made healthcare professionals more approachable, encouraging proactive health discussions and interventions. The dialogues also facilitated the formation of supportive connections, motivating participants to engage with isolated individuals and improve their health. The sustained rural health dialogues effectively reduced loneliness and enhanced health outcomes through increased community engagement and support (Table 1).

**Table 1:** Themes and concepts regarding changes in perceptions and behaviors about loneliness

Theme	Concept
The Existence of Loneliness and Its Impact on Health	Activation of Community Health Dialogues
	Consideration of Community Health as a Whole
	Acknowledgment of Loneliness
	Health Impacts of Loneliness
Motivation to Address Loneliness through a Sense of Security	Ongoing Relationship with Physicians
	Security in Approaching Isolated Individuals
	Community Response to Loneliness
Recognition of the Importance of Community Engagement in Reducing Loneliness	Building Connections through Dialogue
	Encouragement to Participate in Health Dialogues
	Continuity of Dialogue Opportunities
	Awareness of Personal Health Symptoms

**Figure 1:**
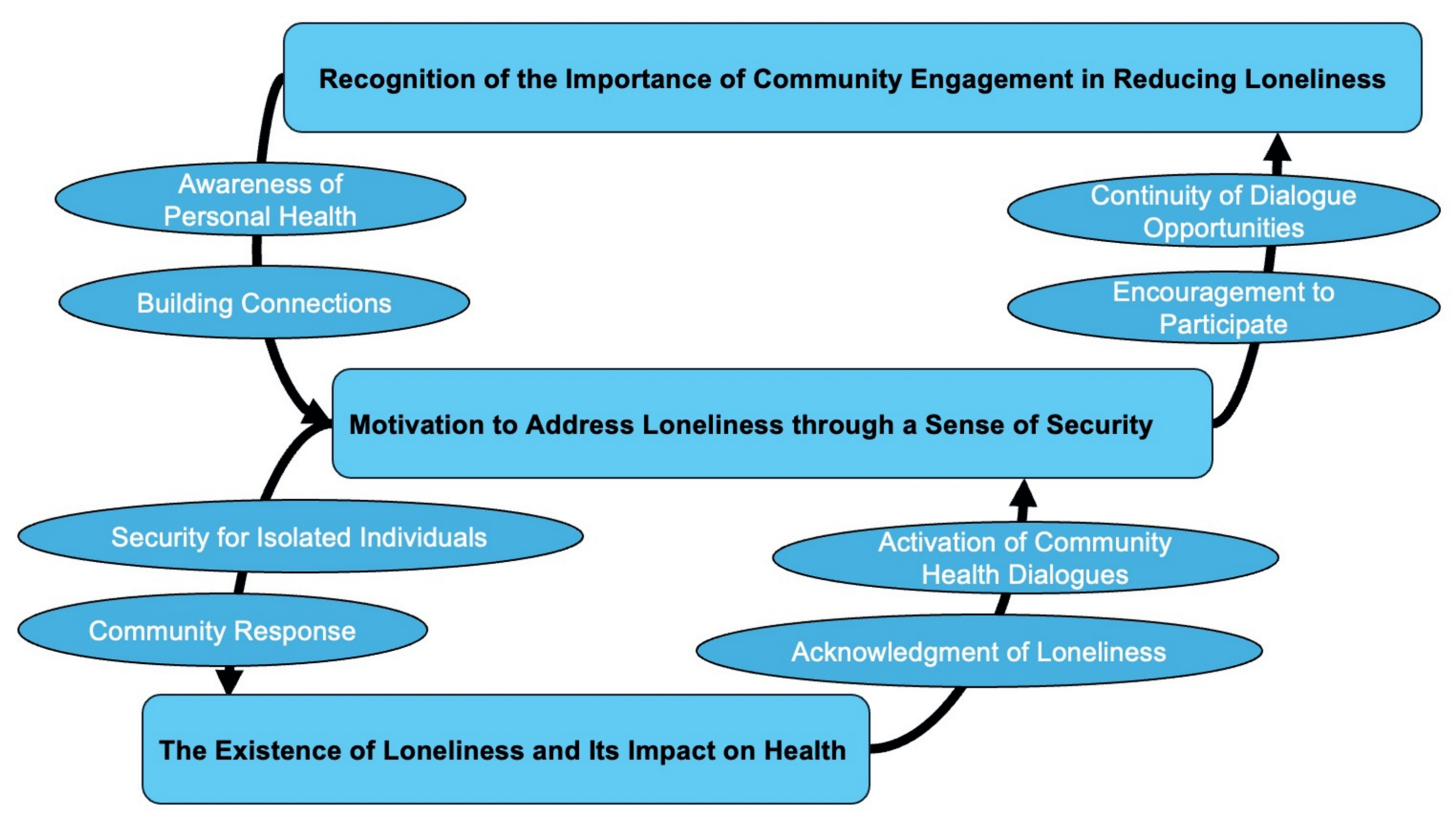
The conceptual figure regarding citizens’ changes in their perceptions and behaviors regarding help-seeking behaviors and loneliness through rural health dialogue Image credit: Ryuichi Ohta

The conceptual figure is the following (Figure 1).

The Existence of loneliness and its impact on health

Activation of Community Health Dialogues

Participants frequently highlighted the invaluable knowledge they acquired through rural health dialogues. Participant 6 noted, "Before these dialogues, I had no idea how much I didn't know about managing my hypertension. Now, I can share this information with my neighbors." This sentiment was echoed by another who said, "It's not just about my health anymore; I feel a responsibility to pass on what I've learned to others in my community" (Participant 11). The dialogues sparked broader conversations within the community, as one participant observed, "We started talking about these health topics at our local gatherings and realized that so many of us had questions that were never addressed before" (Participant 15). This increased communication underscored the significance of the dialogues in fostering a well-informed and connected community, as another participant remarked, "These sessions have opened up lines of communication. We're not just learning from the doctors; we're learning from each other" (Participant 2). Through rural health dialogue, participants realized that activation of dialogue regarding health issues in rural communities deepened their understanding of the community and themselves.

Consideration of Community Health as a Whole

Through the dialogues, participants developed a broader perspective on community health. Participant 11 shared, "I used to think health was just a personal matter, but now I see how interconnected we all are. Each person's health affects the whole community." Another remarked, "These discussions made me realize that we're all in this together. When one of us is healthier, it benefits everyone" (Participant 22). Participants recognized the diverse contributions to community well-being, with one noting, "It's not just the doctors who make a difference. Neighbors helping neighbors, sharing advice and support, that's what keeps us all healthy" (Participant 13). This collective mindset was summed up by a participant who said, "Maintaining our health is a team effort. We all have a role to play, and these dialogues have brought that to light" (Participant 33). The realization of helping each other and having a dialogue about health issues motivated the participants to reconsider community health in depth and broaden their perspectives of health, including loneliness as one health factor.

Acknowledgment of Loneliness

Initially, participants took pride in the strong community ties. Participant 1 stated, "I always thought our community was close-knit and supportive." However, as the dialogues progressed, they began to recognize the presence of loneliness even in rural areas. Another participant admitted, "I was surprised to hear how many people felt isolated, even in our small town" (Participant 24). This realization prompted a deeper reflection on the need to address loneliness. One participant shared, "It's easy to assume everyone is doing fine, but these discussions showed us that's not always the case. We need to pay more attention to our neighbors who might be struggling alone. (Participant 4). Another reflected, "I never realized how loneliness could lead to such serious health problems. We can't ignore this issue if we want to keep our community healthy" (Participant 3). Through rural health dialogue, the participants realized that, even in rural areas where people’s connection was strong, isolation and loneliness were prevalent, affecting their health issues. This newfound awareness underscored the importance of addressing loneliness to prevent health deterioration in isolated individuals.

Health Impacts of Loneliness

Participants observed a noticeable decline in the health of those who had reduced social interactions. One participant remarked, "I saw my neighbor's health deteriorate rapidly after his wife passed away. He stopped coming to community events and seemed to get sicker by the day" (Participant 22). This decline prompted participants to contemplate how the knowledge gained from the health dialogues could be used to improve the health of these individuals. Another participant reflected, "The dialogues have given us tools and information that we can share with those who are isolated. Maybe if we reach out and share what we've learned, we can help them feel better" (Participant 6). Participants discussed the potential for community-driven support, with one stating, "We need to actively check on our neighbors and encourage them to join these dialogues. The information we get here is too valuable to keep to ourselves" (Participant 31). Another participant emphasized, "It's not just about knowing what to do; it's about being there for each other. These health dialogues are teaching us how to support the lonely better and improve their health" (Participant 25). Participation in rural health dialogue taught the participants about isolation and loneliness in rural communities, the importance of sharing information regarding health information, and the disadvantages of isolation and loneliness in health conditions. 

Motivation to address loneliness through a sense of security

Ongoing Relationship with Physicians

Regular interaction with family physicians significantly increased participants' comfort levels with healthcare professionals. One participant shared, "Before these dialogues, I found it intimidating to talk to doctors. Now, after seeing them regularly, I feel much more at ease" (Participant 19). This continuous engagement lowered the barriers to discussing health concerns, making physicians more approachable and accessible. Another participant noted, "The more we interact with the physicians, the more we realize they're just people who want to help us. It’s not as scary to bring up my health issues anymore" (Participant 27). This sentiment was echoed by another who said, "Having these regular sessions builds trust. I used to avoid going to the clinic, but now I feel like I can ask the doctors anything during our dialogues" (Participant 2). Overall, the dialogues fostered a sense of familiarity and trust, as one participant remarked, "It's like we've built a relationship with the doctors. They know us, we know them, and that makes a huge difference in how comfortable we feel about our health" (Participant 21). Continual rural health dialogue mitigated the psychological gap between physicians and rural citizens, which improved rural citizens’ familiarity with family physicians and medical institutions.

Security in Approaching Isolated Individuals

The assurance of being able to consult physicians about health issues motivated participants to reach out to isolated individuals. One participant mentioned, "Knowing that we can always ask the doctors for advice gives us confidence to approach our neighbors who might be struggling" (Participant 6). This sense of security encouraged proactive engagement with those who were lonely and suffering from health problems. Another participant shared, "I used to hesitate to reach out to my elderly neighbor who seemed withdrawn. Now, I feel equipped to offer help because I know I can get accurate information from our health dialogues" (Participant 6). This sentiment was echoed by another participant who said, "Having the doctors' support makes it easier to start conversations about health. We can reassure our isolated neighbors that they are not alone and that help is available" (Participant 23). Overall, this newfound confidence and security empowered participants to take an active role in addressing loneliness and health issues within their community. As one participant concluded, "We no longer feel helpless when we see someone struggling. We know we can make a difference by reaching out and using the knowledge we've gained" (Participant 32). Rural health dialogue secured the participants' ability to approach isolated people regarding their health issues, feeling that they could get support from family physicians whenever they felt difficulties.

Community Response to Loneliness

Participants considered various strategies to mitigate loneliness and promote healthy living in the community. One participant remarked, "We need to find ways to keep our elderly engaged and connected, especially as our population shrinks and households become more isolated" (Participant 10). Another participant added, "It's not just about organizing more events; we need to make sure everyone feels welcome and included" (Participant 19). This awareness highlighted the growing number of elderly individuals experiencing isolation due to population decline and household gaps. Participants emphasized the importance of adapting community structures to address loneliness comprehensively. One participant suggested, "We could set up regular check-ins for those who live alone, maybe through a buddy system or neighborhood groups" (Participant 34). Another proposed, "Using digital tools to connect those who can't easily leave their homes might help bridge some of the gaps" (Participant 1). These discussions underscored the community's commitment to evolving and finding innovative solutions to ensure no one feels isolated. As one participant concluded, "Addressing loneliness requires a collective effort. We need to work together, adapting our community's approach to make sure everyone feels supported and connected" (Participant 29). Through rural health dialogue, the participants realized that they could utilize the resources in rural communities to mitigate the danger of isolation and loneliness. For the utilization, they considered that they should have more dialogue with others in communities to establish better relationships.

Recognition of the importance of community engagement in reducing loneliness

Building Connections Through Dialogue

Participants observed the gradual formation of connections through the dialogues. One participant noted, "At first, it was just a few of us talking, but over time, more people started joining in and sharing their experiences" (Participant 22). Another shared, "It's amazing to see how these small conversations can lead to stronger bonds. People who didn't know each other before are now friends and look out for each other" (Participant 34). Although these connections started small, participants envisioned a positive impact on the health of individuals experiencing loneliness as these connections grew. One participant remarked, "I can already see a difference in some of our elderly members. They seem happier and more engaged now that they have people to talk to regularly" (Participant 7). Another added, "These dialogues are creating a support network. Even if the changes are gradual, they're real and meaningful" (Participant 15). Participants recognized that the dialogues laid the groundwork for a healthier, more connected community. As one participant concluded, "Every connection we make through these discussions is a step towards reducing loneliness and improving our overall well-being. It's a slow process, but it's making a big difference" (Participant 3). Through the rural health dialogue, multiple participants acquired new relationships with various community members in different ways. The new relationships created consecutive connections with others, positively affecting the issues of isolation and loneliness in rural communities.

Encouragement to Participate in Health Dialogues

Participants actively encouraged individuals struggling with health concerns to join the dialogues. One participant shared, "I told my neighbor, who was always worried about her diabetes, to come to the sessions. Just talking about her concerns with others eased her mind" (Participant 22). Another participant added, "Even those who were hesitant at first found comfort in the group. Just showing up and listening made them feel less alone in their struggles" (Participant 45). They noticed that even small steps toward engagement helped alleviate health-related anxieties and fostered a sense of community. A participant observed, "It's incredible how much of a difference it makes when someone starts attending regularly. They become more relaxed and open about their health issues" (Participant 39). Another remarked, "Seeing familiar faces and knowing they share similar concerns creates a bond. It turns health dialogues into a community support system" (Participant 22). Participants emphasized that encouraging others to join was more than just sharing information; it was about building a supportive network. As one participant concluded, "By bringing more people into these conversations, we're not just addressing individual health concerns. We're creating a community where everyone feels cared for and supported" (Participant 9). Rural health dialogue became the opener for health issues discussion in rural communities and regenerating community relationships among citizens, mitigating their anxiety and loneliness caused by social isolation.

Continuity of Dialogue Opportunities

The importance of maintaining regular dialogue opportunities was emphasized, particularly in the context of the COVID-19 pandemic, which has strained many social connections. One participant explained, "During the pandemic, we realized how much we relied on these sessions to stay connected. When they stopped, many of us felt more isolated than ever" (Participant 53). Participants believed that sustained dialogues would reduce loneliness and maintain community health in the long run. Another participant noted, "Regular meetings help us keep in touch and check on each other’s well-being. It's a lifeline for many of us" (Participant 3). The consistency of these dialogues was seen as crucial for building and maintaining relationships. "It's not just about the information we get, but the regularity of seeing each other that makes a difference," Participant 15 remarked. They also saw these opportunities as essential for adapting to ongoing challenges. Overall, participants agreed that continuing these health dialogues was vital for fostering a resilient and connected community as one participant concluded, "Keeping these dialogues going ensures we don't lose the sense of community we've built, especially during tough times like the pandemic" (Participant 20).

Awareness of Personal Health Symptoms

Interactions with physicians during the dialogues made participants more aware of their health symptoms and the risks of ignoring them. One participant stated, "Talking with the doctors made me realize that the aches I was feeling weren't just signs of aging and could be something more serious" (Participant 8). This newfound awareness led participants to understand the importance of sharing health information with others to prevent the progression of undiagnosed conditions. Another participant shared, "I started talking to my friends about what I learned, and we all began paying more attention to our bodies and symptoms we might have overlooked" (Participant 13). These dialogues highlighted the transformative impact of rural health dialogues on addressing loneliness and improving health outcomes. Participants emphasized, "It's not just about learning for ourselves; it's about spreading that knowledge to keep everyone healthier" (Participant 44). The sessions provided a platform for sharing health knowledge and fostering a supportive community environment where individuals felt connected and empowered to take charge of their health. As one participant concluded, "These dialogues have made a huge difference. We feel more connected and better equipped to handle our health concerns, knowing we're not alone" (Participant 31). Rural health dialogue contributed to one of the solutions for loneliness in rural health issues and the realization that participants needed to approach their health conditions honestly before considering their age.

## Discussion

The findings from our study on the continual provision of rural health dialogues underscore the profound impact these interactions have on addressing loneliness and improving overall health in rural communities. The themes identified - the existence of loneliness and its impact on health, motivation to address loneliness through a sense of security, and recognition of the importance of community engagement in reducing loneliness - highlight the multifaceted benefits of these dialogues to overcome rural isolation and loneliness conditions in aging societies.

Activating community health dialogues has been pivotal in fostering a well-informed and connected community. This aligns with previous research emphasizing that community-based interventions promote health literacy and improve health outcomes [17,18]. As this article shows, participants in rural health dialogue realized their present conditions in rural communities and the need to activate community interactions. By sharing health information and personal experiences driven by family physicians, participants gained valuable knowledge and became more proactive in managing their health and supporting others. This community-centered approach is consistent with the findings of other studies that highlight the role of social support networks in mitigating loneliness and enhancing health [2,19,20]. Family physicians’ driven rural health dialogue can initiate interaction among rural citizens regarding health issues approaching isolation and loneliness.

Moreover, the ongoing relationship with family physicians has been instrumental in reducing the psychological barriers that often prevent individuals from seeking medical help. This continuous engagement has fostered trust and made healthcare professionals more approachable, thus facilitating early intervention and management of health issues. Previous literature supports this, indicating that regular and positive interactions with healthcare providers can significantly improve patient satisfaction and health outcomes [21,22]. This research shows that rural citizens became familiar with family physicians and medical institutions. Previous research shows that citizens’ familiarity with medical institutions can mitigate their isolation and loneliness [23]. For the sustainability of communities, continual relationships with family physicians and communities should be driven and enhanced.

Rural health dialogues also played a crucial role in encouraging participants to approach and support isolated individuals within their communities in this research. This proactive engagement is vital in rural settings where healthcare resources are often limited [24]. The sense of security derived from knowing they could consult physicians anytime empowered participants to reach out and offer help, strengthening community bonds [25]. Studies corroborate this by suggesting that community-based health promotion strategies can effectively reduce social isolation and improve mental health [17]. As one community, loneliness and isolation that citizens perceive can be mitigated through interaction with various people, except for their friends and families [3]. In rural health dialogue, participants from various backgrounds in communities can be essential for improving the collectiveness of rural communities and approaching health and isolation issues.

Recognizing the importance of community engagement, participants actively encouraged others to join the dialogues, thereby expanding the support network, fostering a sense of belonging, and leading to the realization of their health conditions and symptoms. This community-driven approach is essential for addressing the broader issue of loneliness, which has been linked to various adverse health outcomes, including cardiovascular diseases and depression [26,27]. By continuously engaging in these dialogues, communities can build resilience and ensure fewer citizens feel isolated [28]. As this article shows, the participants could realize the importance of their health conditions and symptoms more seriously. Previous studies show that caregivers and carers tend to have some severe health issues because of their stress and carelessness to their symptoms [29]. Many community caregivers and carers are stakeholders actively supporting community activities [30]. The continuity of rural health dialogue can activate the notification of their health conditions and drive the sustainability of rural communities.

Our study has several limitations. First, the qualitative nature of the research, while providing in-depth insights, limits the generalizability of the findings. The study was conducted in a specific rural setting in Japan, and the results may not be directly applicable to other rural areas with different cultural, social, and economic contexts. Future research should consider incorporating quantitative measures to validate and extend the findings across diverse rural settings. Second, the study relied on self-reported data, which may be subject to recall and social desirability biases. Participants might have overreported positive outcomes or underreported negative experiences due to the presence of the researchers or the desire to present themselves favorably. Employing objective measures of health outcomes and social interactions in future studies could mitigate this limitation. Lastly, the long-term sustainability and impact of the rural health dialogues were not assessed. While the immediate benefits are evident, evaluating whether these community engagement and health improvements are maintained over time is crucial. Longitudinal studies are needed to understand the enduring effects of these interventions and identify any potential challenges in sustaining them.

## Conclusions

This study highlights the significant impact of rural health dialogues in reducing loneliness and improving health outcomes in rural communities. These dialogues create a supportive network that enhances community cohesion and individual well-being by fostering regular interactions and sharing health information. The findings underscore the critical role of community engagement, the importance of continuous relationships with healthcare professionals, and the proactive approach needed to address loneliness. Policymakers and healthcare providers should consider integrating such dialogues into rural health strategies to combat loneliness and promote healthier communities. Future research should explore these interventions' sustainability and broader applicability across different rural settings. Through continued engagement in rural health dialogues, communities can build more robust social networks, foster resilience, and ensure everyone feels connected and supported.
